# Feedback from human posterior parietal cortex enables visuospatial category representations as early as primary visual cortex

**DOI:** 10.1002/brb3.886

**Published:** 2017-12-18

**Authors:** Yanyan Li, Xiaopeng Hu, Yongqiang Yu, Ke Zhao, Yuri B. Saalmann, Liang Wang

**Affiliations:** ^1^ CAS Key Laboratory of Mental Health Institute of Psychology Beijing China; ^2^ Department of Psychology University of Chinese Academy of Sciences Beijing China; ^3^ Department of Radiology First Affiliated Hospital of Anhui Medical University Hefei China; ^4^ Department of Psychology University of Wisconsin‐Madison Madison WI USA; ^5^ CAS Center for Excellence in Brain Science and Intelligence Technology Shanghai China

**Keywords:** effective connectivity, posterior parietal cortex, primary visual cortex, spatial categorization

## Abstract

**Introduction:**

Categorization is a fundamental cognitive process, whereby the brain assigns meaning to sensory stimuli. Previous studies have found category representations in prefrontal cortex and posterior parietal cortex (PPC). However, these higher‐order areas lack the fine‐scale spatial representations of early sensory areas, and it remains unclear what mechanisms enable flexible categorization based on fine‐scale features.

**Methods:**

In this study, we decoded functional MRI signals and measured causal influences, across visual, parietal, and prefrontal cortex from participants performing categorization based on coarse‐ or fine‐scale spatial information in thirteen healthy adults.

**Results:**

We show that category information based on coarse discriminations was represented in the PPC, in the intraparietal sulcus region, IPS1/2, at an early stage of categorization trials, whereas representations of category information based on fine‐scale discriminations formed later during interactions between IPS1/2 and primary visual cortex (V1). Specifically, when fine‐scale discriminations were necessary, we decoded significant category information from V1 at an intermediate stage of trials and again from IPS1/2 at a late stage. IPS1/2 feedback was critical, because categorization performance improved as causal influence from IPS1/2 to V1 increased. Further, these mechanisms were plastic, as the selectivity of IPS1/2 and V1 responses shifted markedly with retraining to categorize the same stimuli into two new groups.

**Conclusions:**

Our findings suggest that reentrant processing between the PPC and visual cortex enables flexible abstraction of category information.

## 1 INTRODUCTION

Our ability to categorize information based on common aspects or structure in varied sensory experiences is essential for selecting appropriate behavioral responses (Seger & Miller, 2010). Representations of category information have been shown in higher‐order cognitive areas such as the lateral prefrontal cortex (PFC) (Ferrera, Yanike, & Cassanello, 2009; Freedman, Riesenhuber, Poggio, & Miller, 2001; Li, Mayhew, & Kourtzi, 2009) and posterior parietal cortex (PPC) (Freedman & Assad, 2006; Swaminathan & Freedman, 2012). In delayed match‐to‐category tasks, neurons in macaque PFC and PPC often show category‐selective signals after sample stimulus onset and throughout the delay period and test stimulus presentation (Freedman & Assad, 2006; Freedman et al., 2001). It has been reported that the PPC has stronger, earlier, and more reliable category‐related signals than the PFC (Swaminathan & Freedman, 2012), which suggests that category representations can be generated in the PPC.

Because category‐defining information may contain, for instance, high spatial frequency content requiring fine‐scale visual representations, learning new categories may also depend on plasticity in early sensory areas, which has been demonstrated in extrastriate cortical areas (Aizenstein et al., 2000; Goncalves et al., 2015). However, the respective roles of higher‐order and early visual areas and their interactions during categorization remain unclear. One possibility is that category signals are generated in higher‐order areas, such as the PPC, owing to its involvement in both sensory (Bisley, Krishna, & Goldberg, 2004) and higher cognitive functions (Toth & Assad, 2002), whereas early visual cortex, such as the primary visual cortex (V1), strictly represents sensory information on object features and locations that are necessary for extracting category information at higher cortical levels. Alternatively, category signals may be detectable as early as V1 when categorizations rely on fine‐scale features, which would likely involve interactions with higher‐order cortex like the PPC. Such a role for V1 beyond basic sensory processing has been shown in selective attention, working memory, subjective perception, and perceptual learning (Kamitani & Tong, 2005; Li, Piech, & Gilbert, 2008; Roelfsema, Lamme, & Spekreijse, 1998; Super, Spekreijse, & Lamme, 2001; Yan et al., 2014).

To test for category signals in V1 and, if present, the large‐scale network dynamics giving rise to these signals, we used fMRI and multivariate pattern analysis (MVPA) (Haxby et al., 2001) to monitor cortical activity in the PPC, PFC, lateral occipital cortex (LOC), and V1 while participants performed a visuospatial categorization task.

## 2 MATERIALS AND METHODS

### 2.1 Participants

Thirteen healthy adults (seven females; age: 20.70 ± 1.59 years) with normal or corrected‐to‐normal vision participated in the fMRI study. None of the participants had a history of neurological or psychiatric conditions. Informed consent was obtained from all subjects in accordance with guidelines, and the protocol was approved by the Institute of Psychology, Chinese Academy of Sciences. One participant was excluded from the fMRI analysis due to he requested to terminate the scanning session after the first scan.

### 2.2 Experiment design

**Figure 1:**
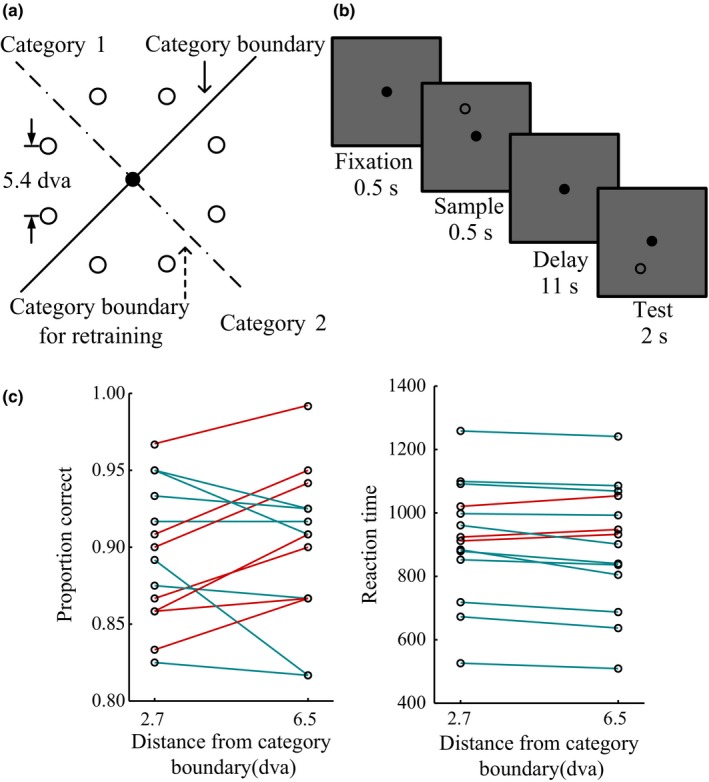
Delayed visual–spatial categorization task and behavioral performance. (a) Circular visual stimuli appeared at one of eight possible locations equidistant from the fixation point. Participants grouped stimuli into two categories defined by an invisible category boundary (solid line). The dotted line is the boundary used when participants retrained to categorize stimuli into two new categories. The boundary lines are shown for illustration only. (b) A sample stimulus was presented for 0.5 s, and after a long delay period (11 s), a test stimulus was presented. Participants reported whether the sample and test stimuli belonged to the same category. (c) Participants’ performance accuracy (left) and average reaction time (RT; right) for the sample stimuli far (6.5 dva) from the category boundary and close (2.7 dva) to the boundary. Red and green lines, respectively, denote increase and decrease from 2.7 to 6.5 dva. Participants’ average RT was shorter for sample stimuli 6.5 dva from the category boundary (compared with 2.7 dva)

In order to assess the role of higher‐order and early visual areas in visuospatial categorization (Figure 1a), we adapted a categorization task previously used in macaque experiments (Crowe et al., 2013). We used a delayed visuospatial categorization (DVSC) task that requires participants to group eight circular stimuli according to learned categorization rules (Figure 1a; category boundary in experiment 1: solid line; experiment 2, after retraining: dotted line). Trials began with the presentation of a square gaze fixation point (each side 0.5 degrees of visual angle [dva]; duration 0.5 s) at the center of the display. Participants were instructed to acquire and maintain their gaze on the fixation point throughout the trial. After the initial fixation period, a circular sample stimulus (0.6 dva in diameter) was presented for 0.5 s at an eccentricity of 7 dva. This was followed by a long delay period (11 s), after which a circular test stimulus was presented (7 dva from the fixation point). The sample and test stimulus positions were selected pseudorandomly from eight possible positions equally spaced around the perimeter of an invisible circle centered at the fixation point. There was 5.4 dva between two adjacent positions). The category boundary in experiment 1 and, after retraining, in experiment 2 was a diagonal line passing through the fixation point at an angle of 45 and 135°, respectively. The positions nearest the boundary were 2.7 dva away from the boundary. If the test stimulus belonged to the same category as the sample, participants were instructed to press the “yes” button on the response device as quickly as possible within the 2‐s test duration. If the test stimulus belonged to the different category, participants pressed the “no” button (Figure 1b). The “yes” button was either on the left or right, which was counterbalanced across the participants. As control trials, a fixation cross instructed participants to simply gaze at the cross (14 s). Before scanning, participants practiced to ensure they would be able to perform the task with greater than 85% accuracy. Participants completed 15 runs (30 trials per position, for a total of 240 trials, and 240 fixation trials) in each experiment. There were four scans totally for each subject (7, 8, 7, 8 runs, respectively).In particular, experiment one and two consisted of two scanning sessions, respectively. Each experiment included 15 runs (a total of 30 runs). Each run was 448s. The sessions performed on separate days with 1 or 2 days separated. A high‐resolution 3D anatomical T1‐weighted scan was acquired from each participant in each scan session.

### 2.3 Visual display

We generated visual displays on a DELL computer using the Matlab Psychophysics toolbox (Psychtoolbox‐3; www.psychtoolbox.org). A 17 × 14 inch liquid crystal display projector outside the scanner room displayed the stimuli on a screen located at the end of the scanner bore. Participants viewed the visual stimuli back‐projected onto the screen at a total length of 12 cm through a mirror attached to the head coil. The screen subtended 45 dva in the horizontal dimension and 37 dva in the vertical dimension.

### 2.4 MRI acquisition

Scanning was performed at the Hospital of Anhui Medical University using a 3‐T GE Discovery scanner. We used foam pads to stabilize the head of each participant and earplugs for ear protection. T1‐weighted images for anatomical localization were acquired using a 3D spoil gradient‐recalled sequence (repetition time [TR] = 7.872 ms; echo time [TE] = 3.06 ms; inversion time [TI] = 400 ms; flip angle = 11°; voxel size = 0.8594 mm × 0.8594 mm × 1 mm; 192 sagittal slices; matrix size = 256 × 256). T2‐weighted images sensitive to blood oxygenation level‐dependent contrasts were acquired using a gradient echo‐planar image pulse sequence (TR = 2,000 ms; TE = 22.6 ms; flip angle = 30°; voxel size = 3.4375 mm × 3.4375 mm × 3.7 mm; 37 axial slices; slice thickness = 3.7 mm; matrix size = 64 × 64).

### 2.5 Data analysis

#### 2.5.1 fMRI data preprocessing

Functional and anatomical images were first analyzed using the FMRI Expert Analysis Tool (part of the FSL package; http://www.fmrib.ox.ac.uk/fsl). Preprocessing of functional images consisted of motion correction, run‐wise linear (1st‐order polynomial) detrending, spatial smoothing (Gaussian kernel, full width at half maximum = 5 mm), and temporal filtering (a nonlinear high‐pass filter with a 90‐s cutoff). Functional images were first registered to the anatomical images and then into the standard (MNI) space. Registration of the functional images with high‐resolution structural images and standard (MNI) space was carried out using affine transformations (Jenkinson & Smith, 2001). Registration from the anatomical images to MNI space was further refined using FNIRT nonlinear registration (Andersson et al., 2007). We performed run‐wise detrending because our data were derived from 15 different runs (two scans), so an assumption of a continuous linear trend across all runs is not appropriate. Global scaling, which was used in a previous Granger causality analysis for fMRI data (Wen, Yao, Liu, & Ding, 2012), was applied to remove the global signal.

#### 2.5.2 Regions of interest (ROIs)

All ROIs that we used are based on probabilistic templates (Mars et al., 2011; Sallet et al., 2013; Wang, Mruczek, Arcaro, & Kastner, 2015; Zhen et al., 2015). Previous studies have shown that the cortical anatomy of V1 is a reliable predictor of the location and retinotopic organization of V1 (Benson et al., 2012). In addition, our recent study revealed that early visual areas were better aligned across subjects within the standard space relative to the higher‐order areas (Wang et al., 2015). Thus, we defined V1 (and occipital areas, V2 and V3, as well as ventral occipitotemporal areas, V4, VOC, and PHC) on the basis of the maximum probability map of visual topography derived from a large subject population (Wang et al., 2015) and LOC based on the maximum probability map for object‐selective regions defined by the contrast between objects and scrambled objects (Zhen et al., 2015). We defined parietal, frontal, and striatum areas on the basis of probabilistic templates of anatomical connectivity and functional interactions (Mars et al., 2011; Sallet et al., 2013; Tziortzi et al., 2014) (Table 1). Note that the PFC, specifically, the dorsolateral PFC, was defined as the combination (logical “or”) of dorsal and ventral BA9/46 (Mars et al., 2011; Sallet et al., 2013; Tziortzi et al., 2014).In particular, V1 included 1,592 voxels, LOC included 10,213 voxels, PPC included 598 voxels, and PFC included 3,838 voxels in the standard space.

**Table 1:** Regions of interest. To obtain regions in the left parietal and dorsal frontal cortex, we flipped the corresponding regions in the right hemisphere across the midline, since the atlases from which our regions were derived only focused on the right hemisphere

ID	Lobe	Region	Abbreviation	MNI (L/R)	Reference
1	Posterior occipital	Primary visual cortex	V1	(−6, −92, −2)/(9, −90, 2)	Wang et al. (2015)
2	Posterior occipital	Secondary visual cortex	V2	d: (−10, −99, 12)/(14, −96, 15)v: (−9, −83, −11)/(10, −81, −8)	
3	Posterior occipital	Third visual complex	V3	d: (−18, −97, 16)/(24, −94, 16)v: (−17, −79, −12)/(18, −77, −11)	
4	Ventral temporal		hV4	(−25, −80, −14)/(26, −79, −12)	
5	Ventral temporal	Ventral occipital cluster	VOC	(−25, −66, −10)/(26, −64, −9)	
6	Ventral temporal	Parahippocampal cortex	PHC	(−27, −52, −9)/(28, −49, −9)	
7	Lateral occipital–temporal	Lateral occipital complex	LOC	(−47, −71, −2)/(48, −68, −3)	Zhen et al. (2015)
8	Superior parietal	Ventral intraparietal area	SPLA	(−30, −41, 53)/(30, −41, 53)	Mars et al. (2011)
9	Superior parietal	Anterior superior parietal cortex	SPLB	(−12, −50, 63)/(12, −50, 63)	
10	Superior parietal	Anterior part of the medial wall of the intraparietal sulcus	SPLC	(−28, −55, 55)/(28, −55, 55)	
11	Superior parietal	Posterior intraparietal sulcus (IPS3)	SPLD	(−19, −63, 53)/(19, −63, 53)	
12	Superior parietal	Posterior intraparietal sulcus (IPS1, IPS2)	PPC/SPLE	(−21, −78, 43)/(21, −78, 43)	
13	Inferior parietal	Parietal opercular region	IPLA	(−49, −25, 30)/(49, −25, 30)	
14	Inferior parietal	Anterior supramarginal gyrus	IPLB	(−53, −32, 44)/(53, −32, 44)	
15	Inferior parietal	Posterior supramarginal gyrus	IPLC	(−50, −44, 43)/(50, −44, 43)	
16	Inferior parietal	Anterior angular gyrus	IPLD	(−46, −55, 45)/(46, −55, 45)	
17	Inferior parietal	Posterior angular gyrus	IPLE	(−37, −67, 39)/(37, −67, 39)	
18	Dorsomedial frontal	Supplementary motor area	SMA	(−10, 4, 59)/(10, 4, 59)	Sallet et al. (2013)
19	Dorsomedial frontal	Presupplementary motor area	preSMA	(−14, 23, 52)/(14, 23, 52)	
20	Dorsomedial frontal	Prefrontal area 9	Area9	(−10, 50, 29)/(10, 50, 29)	
21	Dorsomedial frontal	Frontal polar area 10	Area10	(−16, 58, 4)/(16, 58, 4)	
22	Dorsolateral frontal	Dorsolateral prefrontal cortex	PFC/Area9/46d/v		
23	Dorsolateral frontal	Middle frontal gyrus	Area46	(−31, 48, 11)/(31, 48, 11)	
24	Dorsolateral frontal	Posterior middle frontal gyrus	Area8A	(−30, 9, 52)/(30, 9, 52)	
25	Dorsolateral frontal	Anterior dorsal premotor area	antPMd	(−24, 3, 55)/(24, 3, 55)	
26	Dorsolateral frontal	Lateral superior frontal gyrus	Area8B	(−22, 32, 39)/(22, 32, 39)	
27	Striatum	Limbic target		(−15, 11, −7)/(15, 12, −7)	Tziortzi et al. (2014)
28	Striatum	Executive target		(−18, 10, 5)/(19, 10, 5)	
29	Striatum	Rostral motor target		(−25, 0, 9)/(27, 0, 8)	
30	Striatum	Caudal motor target		(−27, −5, 6)/(28, −5, 7)	
31	Striatum	Parietal target		(−29, −11, 1)/(30, −9, 2)	

MNI (L/R), The Montreal Neurological Institute (MNI) coordinates of the centroids of the left/right region; d, dorsal; v, ventral.

#### 2.5.3 Multivariate pattern analysis

We used pyMVPA for classification analyses (Hanke et al., 2009). We first wrapped the ROIs in standard space into the individual's space. All MVPAs were performed on an individual's space. The multivoxel activity patterns for each stimulus position were analyzed by means of a linear support vector machine (SVM) in combination with a recursive feature elimination (RFE) procedure (De Martino et al., 2008) to estimate the most discriminative voxels. C parameter used in SVM was set −1, which provides automatic scaling of the value according to the norm of the data. We first divided preprocessed functional data into “trials” and labeled them according to stimulus position (here eight positions treated as conditions, 30 trials per position, a total of 240 trials). For all eight positions, correct trials were divided into a training set and a test set using a leave‐one‐run‐out method, which resulted in 15 different splits. The training set (14 runs) was used for deriving maximally informative patterns with the iterative algorithm, and the test set (one run) was only used to assess the performance of classification. The feature selection algorithm (RFE) procedure was performed without an *F* test; especially, we used a RFE to identify those voxels that contributed most strongly to the discrimination of positions. In the first iteration, SVM classifiers were trained and tested including all cortical voxels included in the ROIs defined on the basis of the probabilistic atlases (Sallet et al., 2013; Wang et al., 2015) in a “leave‐one‐out” cross‐validation procedure. Classification accuracy was calculated and 20% of the voxels with the lowest average absolute weights were removed from the feature set. Using only the surviving voxels in the next iteration, new classifiers were again trained and tested until a stopping criterion. The iteration was stopped when performance did not increase in the next 10 iterations. The RFE was performed separately for each time point. Final accuracies at each voxel were computed as the mean over all splits for the test set only. Then, we computed Spearman's correlation between multivoxel activity patterns for different positions in final saved voxels during RFE, that is, similarity values, on the full dataset, with values ranging from −1 to 1. In order to find the true type I error rate, nonparametric Monte Carlo simulations were used to determine the significance of the performances of individual participants; especially, we configured to shuffle the stimuli labels, but only once and only for samples that were labeled as being part of the training set in a particular cross‐validation fold. This is used to perform a cross‐validation analysis under the H0 hypotheses. Next, we assigned the null distribution estimator. The statistical significance threshold was set at *p* < .05. For group level, we used binomial tests to test whether the prediction accuracies of the ROIs were significantly better than chance‐level performance. Importantly, we used several parameters and different feature selection procedures (the RFE procedure or a certain number of the most active voxels in ROIs), and we obtained similar results in each case.

#### 2.5.4 Granger causality analysis

After performing RFE with cross‐validation, the number of voxels most contributing to categorization differed between individuals. To maintain consistent ROIs across subjects, we generated ROIs for Granger causality analyses with spheres of 4 mm radius centered at the voxels with the most sensitivity during the decoding analysis in the individual subject's space. Granger causality analysis was performed in MATLAB using the Granger causality GUI toolbox (http://www.dcs.warwick.ac.uk/~feng/causality.html). Briefly, we estimated the Granger causality for a pair of brain regions during the categorization and fixation trials after removing the first two time points (4‐s) per trial, in order to eliminate the effect of transients. Next, we calculated the average Granger causality across runs (Luo et al., 2013).

#### 2.5.5 Linking Granger causality with behavior

We assessed behavioral performance in each run using reaction time (RT), as RT is sensitive to the stimulus distance from the category boundary (Figure 1c). For each participant, we ranked 15 runs according to RT and sorted them into 11 groups in ascending order, with each group consisting of five neighboring runs. A standardized Granger causality measure was calculated from the “raw” Granger causality values during categorization (GCc) and fixation (GCf) using the following formula: (GCc − GCf)/(GCc + GCf). Next, the standardized Granger causality and behavioral performance in each group were averaged across runs and across participants. The average standardized Granger causality between the IPS1/2 and V1 for each group was plotted as a function of the mean RT z‐score for the group, and the relationship between these two variables was assessed by Spearman's rank correlation.

## 3 RESULTS

### 3.1 Behavioral performance in the delayed visuospatial categorization task

We presented stimuli either 2.7 or 6.5 dva from the category boundary (Figure 1a). Participants categorized sample stimuli that were 2.7 dva (mean 89.5% correct, standard deviation [*SD*] 4.5%) or 6.5 dva (mean 90.0% correct, *SD* 4.9%) from the category boundary with about 90% accuracy. There was no difference in the accuracy of participants for sample stimuli at these different distances from the category boundary (*t *=* *0.496, *p *=* *.628), but there was a significant difference in RT (*t *=* *2.230, *p *=* *.044; Figure 1c).

### 3.2 Stimulus location decoding in higher‐order and early visual areas

Visuospatial categorization requires representations of stimulus locations and category information. Using MVPA, we first investigated the representation of stimulus locations at various stages of visual processing, that is, in the PFC, IPS1/2, LOC, and V1. Although the resolution of spatial representations varies from coarse to fine scales across visual cortex, previous studies have shown that MVPA methods can successfully recover even fine‐scale features from cortical activity sampled at coarser resolutions using fMRI (Harrison & Tong, 2009).

**Figure 2:**
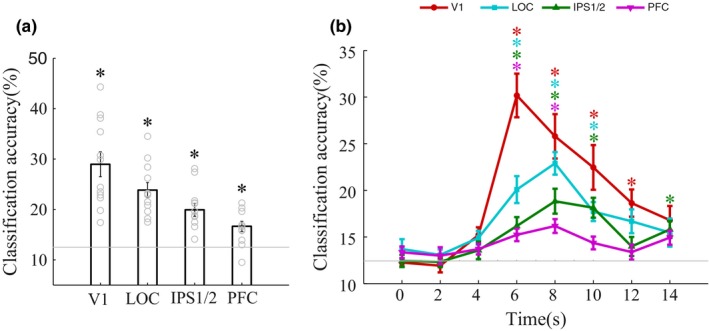
Decoding stimulus location in higher‐order and sensory cortical areas. (a) The classification accuracy of the location of sample stimuli during the delay period (6–10 s) for V1, lateral occipital cortex (LOC), IPS1/2, and prefrontal cortex (PFC). (b) Time‐resolved decoding of individual fMRI time points for V1 (red circles), LOC (turquoise squares), IPS1/2 (green triangles), and PFC (magenta inverted triangles). Note that stimulus location was successfully decoded from both higher‐order and sensory cortex during the delay period. Error bars indicate standard error of the mean, *p <* *.05

For all classifications, ensemble activities pooled over the 6‐ to 10‐s time points during the delay period (using the recursive algorithm) significantly predicted stimulus locations (all *p*‐values <.002), with a prediction accuracy reaching 29%, 24%, 20%, and 17% in V1, LOC, IPS1/2, and PFC, respectively (Figure 2a), where chance‐level performance is 12.5%. There was reliable performance in V1, LOC, and IPS1/2 across participants (*p *<* *.05, binomial test), but not in PFC (*p *=* *.146).

Next, we measured the dynamics of stimulus location representations in the higher‐order and early visual areas by performing the decoding analysis on individual fMRI time points (Figure 2b). Classification accuracy in V1 increased above chance and reached its peak within 6‐s (*t* = 7.56, *p *=* *1.12 × 10^−5^) relative to stimulus onset, and remained significantly elevated until 12 s (all *p‐*values <.00625, corrected for multiple comparisons). In comparison, the classification accuracies of stimulus location in the LOC, IPS1/2, and PFC were lower than in V1 (LOC: *t* = 4.97, *p *=* *4.24 × 10^−4^; IPS1/2: *t* = 5.84, *p *=* *1.13 × 10^−4^; PFC: *t* = 6.81, *p *=* *2.91 × 10^−5^), but nonetheless rose above chance level within 6‐s of stimulus onset (LOC: *t* = 5.26, *p *=* *2.70 × 10^−4^; IPS1/2: *t* = 3.87, *p *=* *2.62 × 10^−3^; PFC: *t* = 4.07, *p *=* *1.85 × 10^−3^) and reached a peak at 8‐s. Classification accuracy remained above chance until 10 s in LOC and IPS1/2, and until 8‐s in PFC, respectively (all *p*‐values <.00625). There was no significant bias in classification accuracies (all *p*‐values >.1) for any stimulus position, in any brain area.

**Figure 3:**
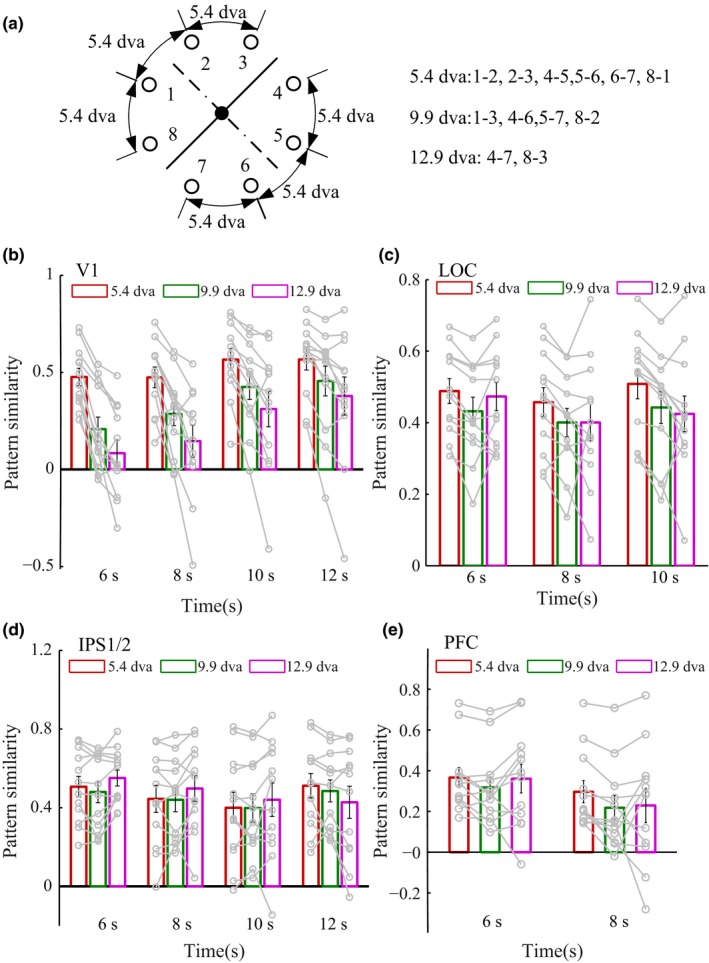
Similarity between multivoxel activity patterns for stimuli separated by different distances in V1, lateral occipital cortex (LOC), IPS1/2, and PFC. (a) Summary of the analysis scheme for pairs of stimuli 5.4, 9.9, and 12.9 dva apart. For example, we computed the 5.4 dva condition across the eight pairs of stimulus positions that were 5.4 dva apart. (b–e) Representation of stimulus positions at time points greatly above chance decoding performance is shown. V1 and IPS1/2 encoded spatial information, and their contribution varied as time elapsed, whereas LOC encoded spatial information similarly throughout the delay period

Finally, we computed the similarity between multivoxel activity patterns elicited by stimuli presented 5.4, 9.9, and 12.9 dva apart. We included stimuli within the same category and in different categories, to eliminate category influence (Figure 3a). There was significant spatial information based on pattern similarity in V1, LOC, and IPS1/2 (all *p‐*values <.05), but not for the PFC (Figure 3b–e). The higher pattern similarity values in V1 for stimuli 5.4 dva apart (compared with 9.9 and 12.9 dva apart) presumably reflect the small receptive field size of V1 neurons.

### 3.3 Category coding in higher‐order and early visual areas

**Figure 4:**
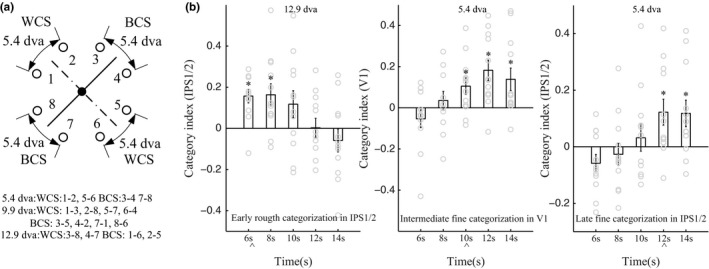
Category coding for higher‐order and sensory areas. (a) Summary of the analysis scheme for stimuli within and between categories. For example, we computed the 5.4 dva category index value by measuring the difference between the within‐category similarities (WCS) for the pairs of stimuli 5.4 dva apart, farthest from the category boundary, and the between‐category similarities (BCS) for the pairs of stimuli 5.4 dva apart, on either side of the category boundary (BCS subtracted from WCS). (b) Coding of category information in V1 and IPS1/2 for coarse and fine discriminations. The IPS1/2 showed early category tuning for the 12.9 dva condition and late category tuning for the 5.4 dva condition. V1 category tuning for the 5.4 dva condition started at an intermediate stage of the delay period. Arrowheads show onset of significant category signals

The above decoding results indicate that both higher‐order and early visual areas are involved in the representation of stimulus locations. If V1, LOC, IPS1/2, and PFC activity can also represent learned categories, then the similarity between multivoxel activity patterns elicited by stimuli within the same category should be greater than that elicited by stimuli in different categories. To test this premise, we first computed six parameters: three parameters for within‐category similarities (WCS) and three parameters for between‐category similarities (BCS). These WCS and BCS parameters represent angles between stimuli of 5.4, 9.9, or 12.9 dva, and each parameter reflects the average of the similarity values calculated for each pair of stimuli at that particular angle of stimulus separation (Figure 4a). For example, we calculated the 5.4 dva BCS parameter by averaging similarities between pairs of stimulus positions that were 5.4 dva apart and crossed the category boundary, and we calculated the 5.4 dva WCS parameter by averaging similarities between pairs of stimulus positions that were 5.4 dva apart and crossed a line perpendicular to the category boundary. We then generated three category indices measuring the difference between WCS and BCS parameters at 5.4, 9.9, and 12.9 dva stimulus separation (i.e., BCS was subtracted from WCS), respectively. Positive category index values indicate more similar multivoxel activity patterns for stimuli within the same category, whereas negative values indicate more similar activity patterns for stimuli in different categories. We found category‐related signals in V1 as well as higher‐order visual areas. Mean category indices were significantly above zero in IPS1/2 at the 6‐ to 8‐s time points for stimuli 12.9 dva apart and again later, at the 12‐ to 14‐s time points, for stimuli 5.4 dva apart. In comparison, V1 only showed significant positive category indices at the 10‐ to 14‐s time points for stimuli 5.4 dva apart (all *p‐*values <.05, Figure 4b). This suggests that category signals based on coarse discriminations first arise in IPS1/2, whereas significant category signals based on fine discriminations first arise in V1.

In this experiment, we defined a particular category boundary to divide stimuli, but it would have been possible to define different boundaries to divide stimuli. To control for nonspecific effects, we determined which of the four possible category boundaries (which exhaustibly divide the eight stimulus positions into two equal groups) results in the greatest difference between average pattern similarities (i.e., greatest category indices) for the four positions on each side of the boundary, for each participant. Notably, for the majority of participants, the actual category boundary was optimal (i.e., it yielded the greatest category indices), and not the other three “irrelevant” boundaries, in IPS1/2 at the 6‐ to 8‐s time points for stimuli 12.9 dva apart, and in V1 at the 10‐s time point for stimuli 5.4 dva apart (IPS1/2, 6‐s: *n *=* *9 of 12, *p *=* *3.92 × 10^−4^; IPS1/2, 8‐s: *n *=* *8 of 12, *p *=* *2.78 × 10^−3^; V1, 10 s: *n *=* *7 of 12, *p *=* *.014, binomial test; Figure 6a and b). Although mean category indices for the actual category boundary were significantly above zero in the LOC at the 8‐ to 10‐s time points, and in the PFC at the 8‐s time point, for stimuli 5.4 dva apart, the actual category boundary was not optimal (i.e., it did not yield the greatest category indices) in LOC and PFC across subjects when compared with the “irrelevant” boundaries, that is, category‐selective signals could not be decoded from LOC and PFC in our DVSC task. This control analysis confirms that IPS1/2 and V1 contained learned category signals.

**Figure 5:**
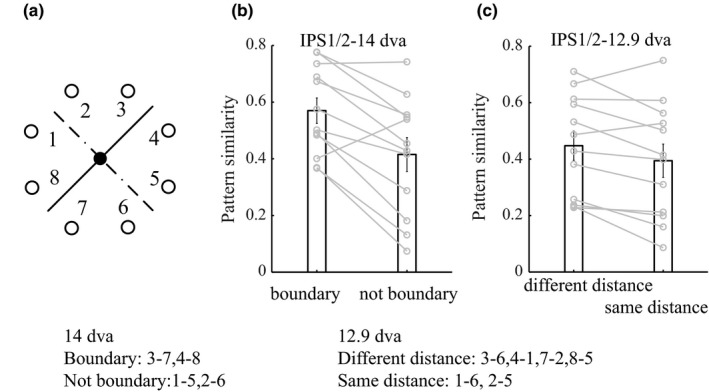
Coarse category coding in IPS1/2 and control for possible attention effects. (a) Summary of the analysis scheme to test categorization and spatial attention. (b) IPS1/2 showed greater pattern similarity for the pairs of stimuli that were 14 dva apart and near the boundary (vs both stimuli far from the boundary), suggesting that early in trials, IPS1/2 signals did not readily distinguish categories when stimuli were near the boundary. (c) Greater pattern similarity for pairs of stimuli located at different distances from the boundary (with one stimulus near the boundary), suggesting that IPS1/2 responses better reflected category processing than spatial attention in our task

In order to further verify that category processing based on coarse spatial discriminations occurred in IPS1/2 at the early stage of trials, we compared the average similarity of multivoxel activity patterns for stimuli 14 dva apart near the category boundary to the pattern similarity for stimuli 14 dva apart but far from the boundary, at the 6‐s time point when IPS1/2 signals had contributed most to categorization performance (Figure 5a). Greater pattern similarity was found for stimuli near the category boundary (*t* = 4.17, *p *=* *1.58 × 10^−3^, paired *t* test), indicating that IPS1/2 signals did not readily distinguish categories early in trials when stimuli appeared near the boundary (Figure 5b). Additional support for this conclusion was derived from the following comparisons of IPS1/2 measures: (i) WCS for stimuli 5.4 dva apart, and BCS for stimuli 5.4 dva apart, all near the boundary; and (ii) BCS for stimuli 14 dva apart near the boundary, and either WCS or BCS for stimuli 5.4 dva apart near the boundary. None of these comparisons were significantly different (all *p‐*values >.1). Taken together, our results demonstrate that IPS1/2 signals mainly coded category information based on coarse visuospatial discriminations at the early stage of trials, whereas V1 signals coded category information based on fine‐scale discriminations at an intermediate/late stage of trials.

### 3.4 Controlling for retinotopy

**Figure 6:**
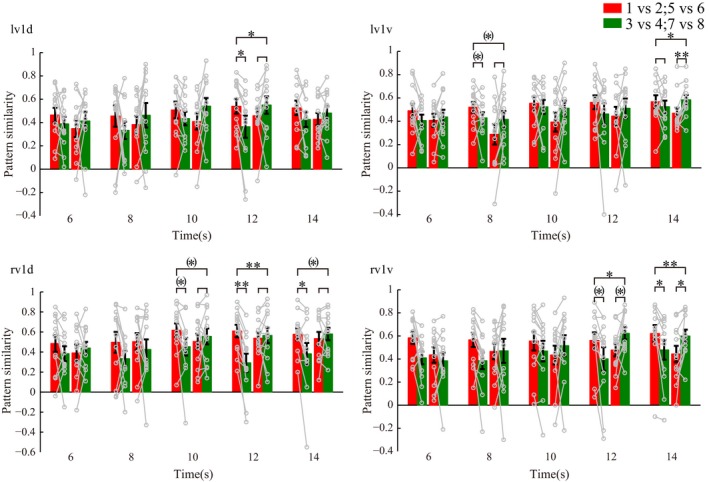
Multivoxel activity patterns in V1 subdivisions depend on stimulus category not just retinotopic location. There were significant or marginally significant interactions between stimulus position groups (group 1: 1 vs 2, 5 vs 6, red bars; group 2: 3 vs 4, 7 vs 8, green bars) and category (experiment 1: two leftmost bars; experiment 2: two rightmost bars, for each time point) at 8‐ to 14‐s time points, suggesting that V1 responses in our task reflected (at least in part) category processing and not just retinotopic organization. Long square brackets indicate interaction between stimulus position and category, and short square brackets indicate within categorization rule effect: (*).05 <  *p* < .1, *.01 <  *p* < .05, *^*^ *p* < .01

To address a possible contribution of retinotopy to our proposed categorical effects in primary visual cortex, we subdivided V1 to exploit the known retinotopy and then measured the relationship between retinotopic and categorical representations (while holding stimulus positions constant but changing their category membership). That is, we constructed a two‐way ANOVA model to test whether retinotopic position and category membership interact with each other in left dorsal V1 (lV1d), left ventral V1 (lV1v), right dorsal V1 (rV1d), and right ventral V1 (rV1v), respectively. An interaction here means that the activity in a particular subdivision of V1 depends on the category to which the stimulus belongs. Specifically, we separated data from the different stimulus positions into two groups based on category membership in experiments 1 and 2. Group 1 includes positions in the upper left (positions 1 vs 2) and lower right (positions 5 vs 6) visual field quadrants, which belonged to the same category in experiment 1 but different category in experiment 2 (Figure 3a). Group 2 includes positions in the upper right (positions 3 vs 4) and lower left (positions 7 vs 8) visual field quadrants, which belonged to the same category in experiment 2 but different category in experiment 1. We averaged similarities between pairs of stimulus positions that were 5.4 dva in the two groups, respectively. To minimize the possible effects of individual subject variations, we used the same areas based on the maximum possibility atlas for each participant. There were significant or marginally significant interactions between stimulus position and category at 8‐ to 14‐s time points (8 s: lV1v, *p* = .069; 10 s: rV1d, *p* = .058; 12 s: lV1d, *p* = .026, rV1d, *p* = .009, rV1v, *p* = .014; 14 s: lV1v, *p* = .045, rV1d, *p* = .053, rV1v, *p* = .008; no significant interaction at 6 s [all *p‐*values >.25]). Post hoc tests (*t* tests, *p*‐values <.05) showed that group 1 is more similar in experiment 1 when its stimuli belonged to the same category, whereas group 2 is more similar in experiment 2 when its stimuli belonged to the same category (Figure 6). These results suggest that V1 responses in our task (at least in part) reflected category processing and not just retinotopic organization.

### 3.5 Controlling for attention

Previous studies have shown that IPS1/2 plays a central role in spatial attention (Bisley & Goldberg, 2010; Petersen & Posner, 2012). In the current study, more similar pattern was found between stimuli along a category line than those away from a category line. One may argue that IPS1/2 effects only represent visual spatial attention, given that attention is a spotlight along a decision line (shaped as a spotlight line). Previous studies revealed that “spotlight” of attention covered a larger section that may be as big further away from the decision line (Muller & Kleinschmidt, 2004). Even if spatial attention existed at the boundary in the current research, it may strongly modulate activity elicited by the stimulus close to the boundary, rather than the stimulus far away, thereby differentiating their activity patterns. Thus, if IPS1/2 effects were due to selective attention, then one might expect greater pattern similarities for stimuli located at the same distance from the category boundary, compared to stimuli located at different distances from the boundary. Thus, we compared the average multivoxel activity pattern similarities for two sample stimuli when one stimulus was near the category boundary (Figure 5a, “different distance”) to the pattern similarities for two stimuli when both were far from the boundary (with stimuli in different categories for all comparisons; Figure 5a, “same distance”). We found that stimuli located at different distances to the category boundary had more similar activity patterns than stimuli at the same distance from the boundary, at the 6‐s time point after stimulus onset when IPS1/2 signals contributed most to categorization performance (*t* = 2.56, *p *=* *.027; Figure 5c). We obtained a similar result when we retrained subjects to use a new category boundary. This suggests that IPS1/2 responses better reflected category processing than spatial attention in our task.

### 3.6 Signal transmission between IPS1/2 and V1

In order to determine how neural signals that encode visual–spatial categories were transmitted between IPS1/2 and V1, we used Granger causality because of the data‐driven nature of this method, and the fact that this method has been successfully applied to fMRI data in investigations of effective connectivity (Hamilton, Chen, Thomason, Schwartz, & Gotlib, 2011; Wen et al., 2012). We found a significantly greater causal influence from IPS1/2 to V1 for categorizations in general compared with the reverse direction from V1 to IPS1/2 (*t* = 4.14, *p *=* *1.64 × 10^−3^; Figure 8a), showing that category processing involves top‐down feedback from IPS1/2 to V1.

Because the MVPA showed V1 signals carried category information during fine‐scale discriminations, we compared the Granger causality for trials in which the sample stimulus was near the boundary (requiring fine discriminations) to trials in which the sample stimulus was far from the boundary (requiring coarse discriminations). We found that V1 had a significantly greater causal influence on IPS1/2 when categorization required fine discriminations relative to coarse discriminations (*p *=* *.037, Figure 8c). This suggests that there are bidirectional interactions between IPS1/2 and V1 when categorization relies on fine discriminations.

To show that interactions between IPS1/2 and V1 relate to the behavioral performance of participants, we first divided task runs into groups based on RT (see Materials and Methods) and then calculated the Granger causal influence between IPS1/2 and V1 for each RT group. If IPS1/2 feedback to V1 improves categorization, then greater Granger causality from IPS1/2 to V1 should correlate with shorter RTs in the DVSC task. Indeed, we found a strong negative correlation between the causal influence from IPS1/2 to V1 and RT (*r* = −.57, *p *=* *.03; Figure 8b). This means that the stronger the causal influence from IPS1/2 to V1, the better the behavioral performance. There was no significant correlation between the causal influence from V1 to IPS1/2 and behavioral performance (*r* = −.10, *p *=* *.77). Considered alongside the MVPA results, this supports the proposal that coarse category processing occurs first in IPS1/2 and, with IPS1/2 feedback, category representations emerge later in V1 when fine discriminations are necessary.

### 3.7 Learning‐based plasticity in IPS1/2 and V1

**Figure 7:**
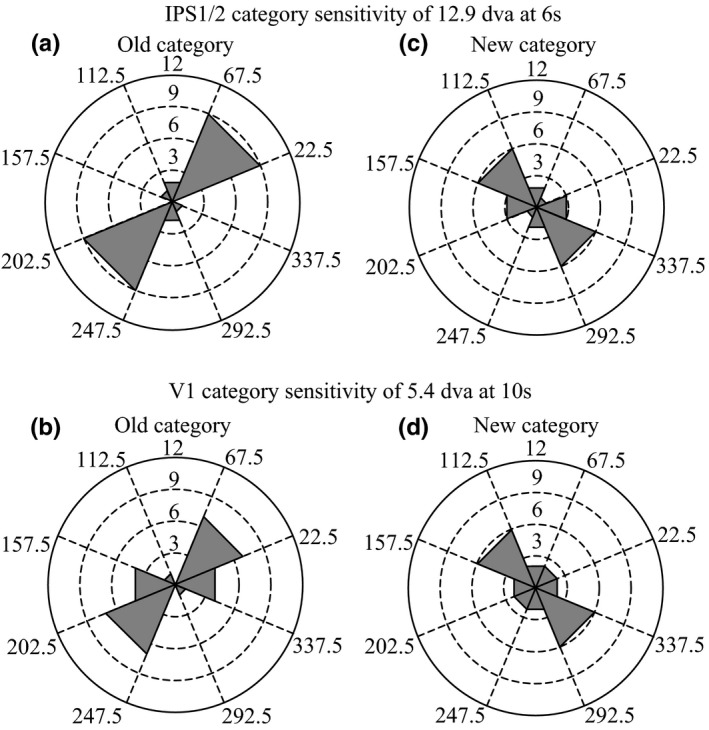
IPS1/2 and V1 category sensitivity before and after retraining. (a and b) Polar plot of the decoded boundary for the 12.9 dva condition at the six‐second time point for IPS1/2, and for the 5.4 dva condition at the 10‐s time point for V1. Extension of gray sector from the center of the plot represents number of participants showing that particular decoded boundary value, before retraining. (c and d) Same format as in a and b, except that data now reflects the retraining of participants to use a category boundary perpendicular to the original one. Note that IPS1/2 and V1 activity showed a learning‐based shift in category sensitivity, but not other areas at any time point

**Figure 8:**
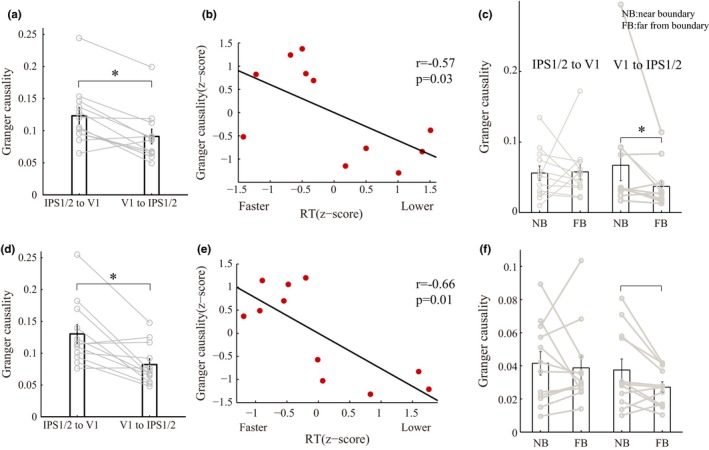
Interactions between IPS1/2 and V1 and their correlation with categorization performance. (a–c) Categorization data for the original category boundary. (d–f) Categorization data for the new category boundary after retraining. (a) Granger causal influences between IPS1/2 and V1 during categorization in general, for the original boundary condition. (b) We grouped task runs according to RT. Population average standardized Granger causality for each group plotted against standardized mean RT for each group. Stronger influence from IPS1/2 to V1 was associated with better categorization performance (faster RT). Linear fits are shown, where *r* is Spearman's correlation coefficient and *p* is the significance level. (c) Granger causal influences between IPS1/2 and V1 when categorizing stimuli near the category boundary and far from the boundary. (d–f) Same format as (a–c), except data now reflect categorization using the new category boundary after retraining

In order to show that the aforesaid categorization mechanisms are flexible, and to further validate that the multivoxel activity patterns in IPS1/2 and V1 were due to the learned categorization rule, we retrained participants recruited in experiment 1 to group the same stimuli into new categories defined by a boundary perpendicular to the original boundary (Figure 1b, dotted line). After retraining, IPS1/2 and V1 selectivity shifted dramatically away from the previous category boundary, to now reflect the new category information. Consistent with the results from experiment 1 (Figure 7a and b), the multivoxel activity patterns of the participants in experiment 2 were best classified according to the new category boundary, and not the old boundary, in IPS1/2 and V1 (IPS1/2, similarities for stimuli 12.9 dva apart, at 6‐s: *n *=* *6 of 12, *p *=* *.054; V1, similarities for stimuli 5.4 dva apart, at 10 s: *n *=* *6 of 12, *p *=* *.054; binomial test; Figure 6c and d). Additionally, in experiment 2 with the new category boundary, there was significantly greater top‐down Granger causal influence from IPS1/2 to V1 for categorizations in general (compared with influence from V1 to IPS1/2; *t* = 3.74, *p *=* *3.26 × 10^−3^; Figure 8c), and stronger influence from IPS1/2 to V1 was associated with better categorization performance (faster RTs; *r* = −.66, *p *=* *.01; Figure 8d). Together, these results indicate a profound learning‐based plasticity of category representations in IPS1/2 and V1, in which early IPS1/2 signals contained coarse category information, whereas later V1 signals contained category information pertaining to finer‐scale discriminations. The strong correlation between the top‐down IPS1/2 to V1 influence and behavioral results further suggests that the IPS1/2 and V1 abstract experience‐dependent category representations cooperatively, reaffirming our fMRI decoding results.

### 3.8 Decoding activity patterns in other brain areas

After identifying IPS1/2 and V1 category representations in the above analyses, we repeated the same MVPA procedure for 27 other regions of interest, including two areas in the occipital cortex, three areas in the ventral occipital–temporal cortex, nine areas in the parietal cortex, eight areas in the dorsal frontal cortex, and five regions in the striatum, in order to test whether other regions may carry category information. None of these areas showed reliable category signals during either coarse or fine discriminations.

## 4 DISCUSSION

Our results indicate that IPS1/2 and V1 are important for encoding category information during the delay period of the delayed match‐to‐category task. These areas played distinct roles in categorization: IPS1/2 contained category information at an early stage of the delay period during coarse discriminations and at a late stage when fine discriminations were necessary, whereas V1 contained category information at an intermediate stage during fine discriminations. When category signals emerged in the IPS1/2, the IPS1/2 provided feedback to V1: The stronger the influence from IPS1/2 to V1, the better the categorization performance. Moreover, the IPS1/2 and V1 category representations reorganized when participants learned new categories. These findings demonstrate the learning‐based plasticity of visuospatial category representations in human IPS1/2 and V1 as well as the flexibility of IPS1/2‐V1 interactions, which enable the abstraction of new category information from multiple spatial scales.

Previous studies in macaques have shown category‐selective activity of neurons in the PPC, particularly in the lateral intraparietal area (LIP) (Freedman & Assad, 2006; Swaminathan & Freedman, 2012). LIP neurons were first shown to respond to categories defined by motion direction, and more recent work suggests that LIP neurons can represent learned associations between a broad range of visual stimuli (Fitzgerald, Freedman, & Assad, 2011). Macaque LIP exhibits a number of response characteristics similar to the human IPS1/2 region (Konen & Kastner, 2008; Szczepanski, Konen, & Kastner, 2010). Our study shows that IPS1/2 also represents category information like LIP, and goes beyond previous work by showing category information can be decoded as early as V1.

Although the classical view of V1 is as a purely sensory area, recent fMRI results have revealed that contextual information as well as internal states can influence V1 responses (Muckli, 2010). Available evidence suggests that V1 contributes to selective attention, working memory, subjective perception, and perceptual learning (Kamitani & Tong, 2005; Li et al., 2008; Roelfsema et al., 1998; Super et al., 2001; Yan et al., 2014). A recent study suggests that perceptual learning may occur on a conceptual level, similar to object category learning (Wang et al., 2016). Because these, and other, cognitive and perceptual operations often require processing of fine‐scale information, it may be necessary to draw upon the high‐resolution visual map in V1.

When learning new categories, interactions between early sensory and higher‐order cortical areas would allow the integration of information from multiple visual maps at different spatial scales, to build stable category representations. Consistent with this, learned category information has been shown at multiple cortical levels, including the PFC (Ferrera et al., 2009; Freedman et al., 2001; Li et al., 2009), PPC (Braunlich, Gomez‐Lavin, & Seger, 2015; Freedman & Assad, 2006; Sarma, Masse, Wang, & Freedman, 2016; Swaminathan & Freedman, 2012), extrastriate visual cortex, where activity in V3 and V3A changed with perceptual category learning (Aizenstein et al., 2000; Goncalves et al., 2015) and now V1 in our study. Feedback signals have been demonstrated between each of these cortical levels (Buschman & Miller, 2007; Chen et al., 2014; Moldakarimov, Bazhenov, & Sejnowski, 2014; Piech, Li, Reeke, & Gilbert, 2013; Saalmann, Pigarev, & Vidyasagar, 2007), and computational modeling work suggests that ultimately the feedback can result in learning in V1, for instance, from plasticity in top‐down inputs or top‐down gating of lateral interactions in V1 (Chen et al., 2014; Moldakarimov et al., 2014; Piech et al., 2013).

How do the interactions between IPS1/2 and V1 give rise to category representations based on fine‐scale features? One possibility consistent with our data is that the sample stimulus causes V1 neurons with receptive fields at the stimulus location to undergo short‐term plasticity or to show persistent activity (as spiking or oscillatory activity), that is, to retain a memory of the stimulus location. Because PPC neurons have relatively large receptive fields (e.g., in macaque LIP, 10 dva receptive fields are common), this may limit their ability to generate category‐selective signals based on fine‐scale information, initially giving rise to only weak category‐selective activity. Nonetheless, these PPC neurons can provide some feedback on categories to V1, possibly via extrastriate cortex. The feedback signals would have their greatest effect on those V1 neurons that retained a memory of stimulus location (because the stimulus had previously potentiated synapses or depolarized neurons, bringing them closer to action potential threshold). Thus, these V1 neurons would show increased excitability, reflecting category‐selectivity built on fine‐scale information, which could be transmitted to PPC to refine its own category representation. Another possibility does not require V1 to maintain information on the location of the sample stimulus. Instead, high spatial resolution may still be achievable when there is partial overlap of the large receptive fields of multiple PPC neurons. Although any one of these PPC neurons would be insufficient, their combined output may have sufficient resolution when read out in V1, leading to enhanced responses of a select group of V1 neurons. In this case, V1 category signals would be a direct result of PPC feedback.

Although a number of studies have demonstrated that the PFC can encode category information (Crowe et al., 2013; Freedman & Assad, 2006; Freedman et al., 2001; Li et al., 2009; Swaminathan & Freedman, 2012), we only found moderate category‐related modulations of PFC multivoxel pattern activity, which did not survive the control for nonspecific boundary effects. In comparison, IPS1/2 showed reliable and robust category signals during our DVSC task. One explanation is that our task was not sufficiently demanding to warrant significant engagement of the PFC. That said, our results are consistent with prior nonhuman primate work, in which stronger and more reliable category signals were found in the PPC relative to PFC (Swaminathan & Freedman, 2012).

In addition to category processing, the PPC plays a role in selective attention (Bisley & Goldberg, 2010; Petersen & Posner, 2012) and movement planning (Buneo & Andersen, 2006; Cui, 2014). However, it is unlikely that spatial attention or motor intention confounded the category signals we reported in IPS1/2 (Bisley & Goldberg, 2010). First, our control analysis showed that IPS1/2 activity in our DVSC task better reflected category processing than spatial attention to visual stimuli near the category boundary. Second, evidence from nonhuman primate studies suggests that PPC neurons can exhibit category signals even when stimuli are presented outside their receptive field (Freedman & Assad, 2009). This suggests that PPC neurons can independently encode spatial and nonspatial information. Third, although there may be an anticipation that behavioral responses will either be “yes” or “no,” our results cannot be explained only by an intention embedded within the motor planning system because there is an explicit dissociation of the categorization of the sample stimulus from the participant's response (which can only be precisely planned after the test stimulus) in our task. Finally, all category effects in our study reflect a contrast between different stimuli, to exclude a common intention/anticipation effect.

It is noteworthy that the paradigm we used cannot exclude the possibility of attention priming effects, whereby seeing one location in one spatial location primed the activation of the other locations to some degree. Additionally, although fMRI furnishes time‐series data with high spatial precision, two features are especially problematic: (i) poor time resolution; and (ii) BOLD responses reflect delayed neural activity due to a convolution with an HRF, and may have significant interregional variability. Thus, the current applications of Granger causality to fMRI should be treated cautiously and require carefully chosen experimental paradigms. Finally, previous visual working memory studies have been argued that activity in early visual area is likely a top‐down priming signal generated in anticipation of the upcoming probe stimulus to facilitate the comparison between the remembered sample stimulus and the probe stimulus (Lui & Pasternak, 2011; Serences, 2016). That is, the location of the sample stimulus and its surrounding locations belonging to the same spatial category could be activated when it was near the end of the delay period. Thus, increased WCS may be found in V1 at 10s relative to 8s and in IPS1/2 at 12 s relative to 10s (fine discrimination signal found in V1 at 10s, in IPS1/2 at 12a). However, no significant differences were found in these analyses (both *p*s >.15). Another possible explanation is perhaps that there were top‐down rehearsals in V1 to identify which category the remembered sample stimulus was belonged to. Due to poor time resolution of fMRI, further studies, such as intracranial electroencephalography studies, are required to test these possibilities. In summary, our study suggests that category processing based on coarse discriminations occurs first in the higher‐order cortical area, IPS1/2, which is ideally positioned to transform sensory information into more abstract representations. Feedback from IPS1/2 then enables category processing based on fine discriminations in early visual areas, including V1, which is well suited for processing fine‐scale features that often aid in categorization.
